# Natural Keratin and Coconut Fibres from Industrial Wastes in Flame Retarded Thermoplastic Starch Biocomposites

**DOI:** 10.3390/ma12030344

**Published:** 2019-01-22

**Authors:** Sebastian Rabe, Guadalupe Sanchez-Olivares, Ricardo Pérez-Chávez, Bernhard Schartel

**Affiliations:** 1 Bundesanstalt für Materialforschung und-prüfung (BAM), 12205 Berlin, Germany; bernhard.schartel@bam.de; 2 CIATEC, A.C. Center of Applied Innovation in Competitive Technologies, Guanajuato 37545, Mexico; gsanchez@ciatec.mx (G.S.-O.); rperez@ciatec.mx (R.P.-C.)

**Keywords:** biomaterials, biodegradable, calorimetry, composites, flame retardance

## Abstract

Natural keratin fibres derived from Mexican tannery waste and coconut fibres from coconut processing waste were used as fillers in commercially available, biodegradable thermoplastic starch-polyester blend to obtain sustainable biocomposites. The morphology, rheological and mechanical properties as well as pyrolysis, flammability and forced flaming combustion behaviour of those biocomposites were investigated. In order to open up new application areas for these kinds of biocomposites, ammonium polyphosphate (APP) was added as a flame retardant. Extensive flammability and cone calorimeter studies revealed a good flame retardance effect with natural fibres alone and improved effectiveness with the addition of APP. In fact, it was shown that replacing 20 of 30 wt. % of APP with keratin fibres achieved the same effectiveness. In the case of coconut fibres, a synergistic effect led to an even lower heat release rate and total heat evolved due to reinforced char residue. This was confirmed via scanning electron microscopy of the char structure. All in all, these results constitute a good approach towards sustainable and biodegradable fibre reinforced biocomposites with improved flame retardant properties.

## 1. Introduction

The recycling of industrial wastes is an economically interesting and environmentally friendly approach towards sustainable material resources. For an efficient manufacturing and usage lifecycle, it is necessary for the processing plant to be in the vicinity of the material source. The natural fibre wastes investigated in this paper are derived directly from Mexican industrial sectors and processed in Mexico, to reduce transport and storage effort to a minimum.

In order to create a completely biodegradable biocomposite obtained from renewable resources, synthetic fillers such as carbon or glass fibres need to be replaced and a biopolymer must be used as matrix [1,2,3]. An inexpensive and ecological and thus sustainable, alternative is found in natural fibres derived from industrial process wastes. These may come from plant-based sources like bast fibres as well as animal sources [4]. Vegetal fibres are composed of cellulose, hemicellulose and lignin, while animal-based fibres consist mainly of proteins, posing a valuable nitrogen source. In polymer composites, the main application of natural fibres has been as mechanical reinforcement [5]. The properties of the natural fibre reinforced polymer composites depend on different variables including fibre type, aspect ratio (length/width), content, modification method, interaction with the polymer matrix and processing conditions [6]. For example, sisal fibre lengths greater than 10 mm (20 and 30 mm) improve the mechanical properties of sisal/polypropylene composites. The samples were obtained by hot pressing and the composites with 30% mass fraction of fibre exhibited tensile, flexural and impact strength greater than polypropylene. The authors mentioned that, when the fibre mass fraction is added at low content, the fibres may act as filler leading to a reduction in the strength of the composite. In addition, they compered their results with sisal-polystyrene composites, in which the low tensile strength was attributed to the random orientation of the short fibres [7]. Similarly, the mechanical performance of abaca, jute and flax fibres on polylactic acid (PLA) biocomposites was evaluated using 30 wt. % fibre content. Jute fibres showed better tensile strength than abaca and flax fibres, while abaca fibres increased the Charpy notched impact strength (total amount of energy that a material is able to absorb) more than jute and flax fibres did. The later results point out that, the natural fibre type has a strong influence on the mechanical properties of the composites. There are several types of natural fibres, which differ regarding their chemical structure [8]. Otherwise, reinforced PLA/abaca fibre biocomposites, obtained by extrusion and injection moulding with 30 wt. % abaca fibre content, demonstrated a tensile modulus improved by a factor of 2.4, flexural modulus improved by a factor of 1.20 and impact strength 2.4 times greater than unreinforced PLA. According to the morphology of the composites, abaca fibres seem to be well coated with PLA matrix ascribed to the surface roughness of abaca fibre. The author suggested that reinforcing PLA with natural fibres leads to good chemical bonding on the interphase [9].

Regarding animal-based fibres, they are characterized by different properties than vegetal fibres. For instance, wool keratin fibres possess surface toughness, flexibility and a high aspect ratio and are less hydrophilic than vegetal fibres. Feather keratin fibres have a hollow structure and thus present extremely low fibre density and a low dielectric constant (k). The potential of chicken feather keratin fibres has been used to produce a low-k dielectric composite for electronic applications. The composites were obtained using acrylated epoxidized soybean oil and keratin feather fibres. The hollow keratin fibres were not filled by resin infusion and the composite retained a significant volume of air in the hollow structure of the fibres. The resulting dielectric constant (k) of the composites depend on the keratin feather fibre volume fraction due to the retained air. The values obtained in this work were significantly lower than the conventional silicon dioxide or epoxy or polymer dielectric insulators [10]. In addition, feather keratin fibres have been used as a reinforcement agent in polymer composites. As an example, 20 wt. % of feather keratin fibres were added to high density polyethylene (HDPE). Temperature, time and speed during compounding step were studied. The authors reported the optimal conditions of the processing to maximize fibre-polymer interactions and minimize degradation of feather keratin fibres. The composite showed improved stiffness and lower density than virgin HDPE [11]. 

Thermoplastic starch is derived from potatoes, corn or other cereals and consists mainly of amylose and amylopectin. Due to its high sensitivity towards hydrolysis as well as its poor dimensional stability and mechanical properties, the starch phase is blended with a biodegradable polyester [12]. This upgrades thermoplastic starch into a biodegradable material interesting for various applications. Commercially available starch blends are typically used as food packaging material or in disposable and compostable items and films [13]. The increased demand for biodegradable materials makes it necessary to research further potential application areas, for example in the transport, construction, electric and electronic industries. For the utilization of natural fibre composites in these sectors it is essential to assess their fire retardancy [14]. Thermoplastic starch has demonstrated good flame retardancy effects through the combination of coconut fibres and aluminium trihydroxide; the heat release rate, fire growth rate and total fuel release were reduced significantly. These results were explained by the increase in carbonaceous char accompanied by the reduction of carbon content in the pyrolysis products. Moreover, using coconut fibres on thermoplastic starch allowed the reduction of the aluminium trihydroxide content. The results offer a foundation for reducing the content of a traditional flame retardant additive by adding natural fibres to thermoplastic starch biocomposites [15]. The thermoplastic starch- blends, one of which was used as a polymer matrix in this publication, were shown to decompose to a satisfying degree in a composting environment and generally achieve major degradation rating in environmental exposure [16] Degradation in anaerobic conditions showed a decomposition similar to cellulose filter paper, measured by decrease of volatile solids [17]. In general, these thermoplastic starch-blend products are made with regards to compostability in order to reduce organic waste landfill disposal and thus biogas production, the main hazard of organic waste. 

Flame retardants based on phosphorus are widely used to replace halogen-based additives, which exhibit toxicity and pose environmental risks [18,19,20]. Several flame retardants based on phosphorus are available on the market [21,22,23,24]. Phosphate compounds have been used extensively in order to reduce the flammability of cellulosic materials. For example, the effect of ammonium polyphosphate (APP) as a flame retardant additive, in combination with natural fibres, has been investigated for various polymer matrices. Polypropylene, polyurethane and thermoplastic starch using wood flake and corn shell in combination with APP exhibited flame retardancy improved up to the self-extinguishing V0 rating. From this research, it was observed a charring behaviour of polysaccharides and polyurethane in presence of APP. These results demonstrated the efficiency of APP as a flame retardant additive for biopolymer systems [25].

The present work focuses on the processability and properties of natural fibre biocomposites with an emphasis on flame retardant behaviour with and without APP as a flame retardant. The synergism between natural fibres and APP in flammability and burning behaviour under forced flaming conditions is exposed and explained. One of the main objectives of this work is to reduce the high content of APP added to conventional flame retardant composites by taking advantage of waste materials but maintaining or even improving the mechanical and flame retardant properties of the conventional composites. Moreover, to the best of our knowledge, the use of natural keratin and coconut fibres derived from industrial wastes in combination with halogen free ammonium polyphosphate to produce fire retardant polymer composites based on biodegradable thermoplastic starch has not been reported previously.

## 2. Materials and Methods 

Thermoplastic starch (TPS), Mater-Bi^®^ EF05B, 100% compostable biopolymer, was purchased from NOVAMONT SpA (Novara, Italy). Keratin fibres (KF) were recovered as waste from the beamhouse stage of a Mexican tannery. Coconut fibres (CF) were acquired from COPEMASA Co. (Tecomán, Mexico) as waste from the husks of coconut fruits. Ammonium polyphosphate (APP), trade name Exolit AP-422, was supplied by Clariant GmbH (Wiesbaden, Germany), with 31–32 wt. % phosphorus content and a particle size of D50 < 17 µm.

The KF were obtained as a by-product of waste hair from the beamhouse stage after liming during processing at a tannery. KF treatment was carried out using a tannery test drum. The process consists of two main steps, deliming and degreasing. For deliming, ammonium sulphate and sodium bisulphite were added at a concentration of 0.5% with respect to the weight of the waste hair. The drum was rolled for 2 h after rinsing. The process was repeated until a neutral pH was achieved (7.5–8.5). The degreasing step was similar but used a tensoactive product at 1% concentration with respect to waste hair weight. Then the KF were drained and dried in an oven at 75 °C for 12 h. Finally, KF were sieved through a 2 mm mesh.

The CF were pulverized twice in a 7.5 HP ASF P200 machine from Alimentos y Servicios Funcionales, S.A. de C.V. (Mexico City, Mexico), first through a 5 mm mesh and then through a 1 mm mesh. Afterward the pulverized coconut fibres were sieved through a #60 mesh (250 µm). Before the compounding step, the CF were dried at 80 °C for 12 h.

TPS composites were obtained by extrusion in a twin-screw Leistritz Micro 27 extruder from Leistritz Advanced Technologies Corp. (Nuremberg, Germany), L/D = 32, with a diameter of 27.0 mm and 8 heating zones, using counterrotating intermeshing mode. The compounding was carried out under the following temperature profile: 130/135/135/140/145/150/145/140 °C (from feed to die). In order to improve the dispersion of fibres and flame retardant additives, the composites were extruded twice at a rotational speed of 120 rpm. The TPS and composites were dried before extrusion at 105 °C in a model 30 low pressure dryer from Maguire Products Inc. (Des Moines, IA, USA), with 80 psi (0.5516 MPa) maintained during heating. After each extrusion step the composites were granulated in a 7.5 HP Paganí granulator (Pagani, Mexico City, Mexico) using a screen with a 5 mm mesh. Table 1 describes the composition of the investigated materials.

Specimens for UL 94 testing, oxygen index (OI) determination, cone calorimeter and mechanical tests were obtained by injection moulding in a Milacron TM55 model machine from Milacron LLC, Karnataka, India, with 4 heating zones. The injection moulding conditions were used as follows: 145/150/155/155 °C temperature profile (from feed to die), 95 mm/s injection speed, 110 bar injection fill pressure and 45 s cooling time. Before injection moulding all TPS composites were dried under the same conditions as those used for extrusion.

Morphology analysis was performed in a JEOL JSM-7600F field emission scanning electron microscope (SEM, JEOL, Ltd., Akishima, Japan), using fractured samples of the composites. Elemental analysis was carried out by the incorporated energy dispersive spectroscopy (EDS) over the scanned area.

Mechanical tests were carried out using a tensile Instron 5565, from Instron (Norwood, MA, USA), machine at 50 mm/min crosshead speed following the ASTM D638 standard, with sample dimensions according to type I and a thickness of 3.1 ± 0.1 mm. Izod impact resistance was evaluated according to the Izod notched ASTM D256 standard, using samples 64 mm × 12.7 mm × 12.7 mm in size. 

Rheological analysis was performed in a strain-controlled Ares-G2 rheometer, from TA-Instruments (New Castle, DE, USA), using parallel plates (25 mm diameter) at 165 °C and a gap of 1.0 mm under Small Amplitude Oscillatory Shear flow (SAOS); the tests were performed in a linear viscoelastic regime.

Thermal analysis of the samples was conducted on a Netzsch TG 209 F1 Iris (Selb, Germany). Portions of 5 mg of a specimen were pyrolyzed under nitrogen at a heating rate of 10 K/min.

Burning behaviour under forced flaming conditions was analysed in a Fire Testing Technologies cone calorimeter (East Grinstead, UK). The specimens (100 mm × 100 mm × 3.1 mm) were conditioned at 23 °C and 50% RH for at least 48 h prior to measurement, wrapped in an aluminium tray and measured at an external heat flux of 50 kW/m^2^ and a distance of 35 mm from the cone heater, to avoid contact between the sample and the spark igniter or cone heater in case of strong intumescence. Heat flux impact uniformity on the specimens was shown to remain very similar up to a distance of 25 mm [26]. Samples were measured in duplicate unless the results deviated by more than 10%. 

The flammability of the specimens was characterized by the limiting oxygen index (OI) and classification in the UL 94 test. Oxygen index measurements were conducted according to ISO 4589 with a sample size of 100 mm × 6.5 mm × 3.1 mm and UL 94 classifications were achieved according to IEC 60695-11-10 with a sample size of 127 mm × 12.7 mm × 3.1 mm. Specimens for flammability tests were conditioned similar to cone calorimeter specimens. 

## 3. Results and Discussion

### 3.1. Morphology

**Figure 1:**
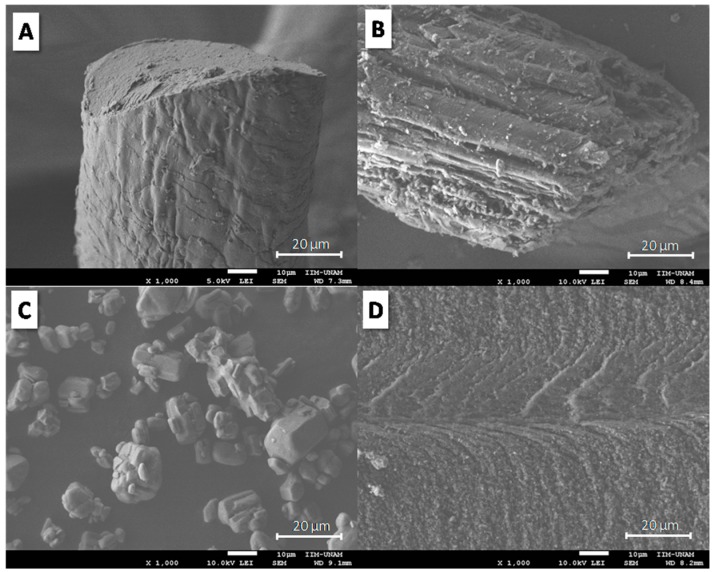
SEM micrographs of (**A**) KF, (**B**) CF, (**C**) APP and (**D**) TPS.

The morphology of keratin and coconut fibres, APP additive and the composites was studied by Scanning Electron Microscopy (SEM). Figure 1 displays the morphology of keratin (Figure 1A) and coconut (Figure 1B) fibres, as well as APP (Figure 1C) and TPS (Figure 1D). According to Figure 1A, keratin fibre shows a cylindrical shape, with a diameter of approximately 80 µm; overlapped layers on the keratin fibre surface are clearly observed. Coconut fibre morphology (Figure 1B) presents a width of approximately 80 µm and consists of microfibres of different widths (approximately 10–15 µm) [27]. Figure 1C reveals APP morphology. According to the APP micrograph, agglomerates, irregularly shaped particles and different particle sizes (approximately 5–18 µm) are observed. Figure 1D displays the morphology of the fractured surface of the TPS matrix. The TPS micrograph shows well-defined fractured planes, characterized by thin layers oriented toward the direction of fracture. According to Figure 1D, TPS morphology exhibits a ductile fracture surface.

**Figure 2:**
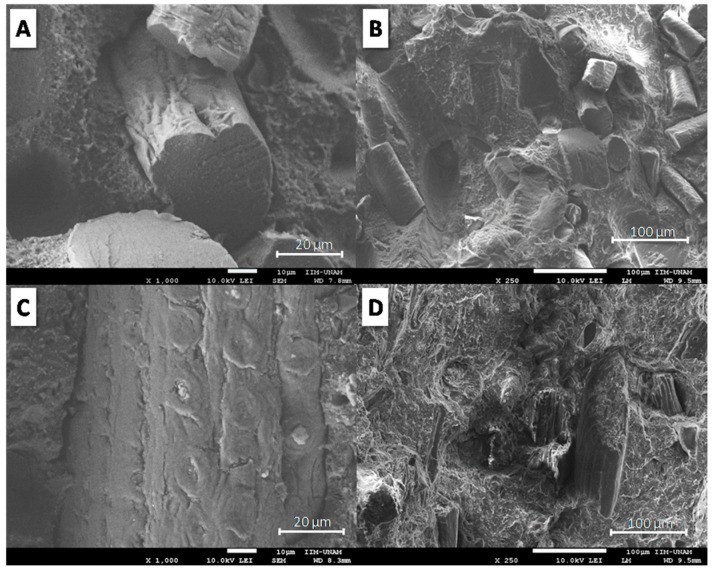
SEM micrographs of the fractured surface of TPS/20KF (**A**,**B**) and TPS/20CF (**C**,**D**) at two different magnifications.

Figure 2 shows the morphology of fractured surfaces for TPP/20KF (Figure 2A,C) and TPS/20CF (Figure 2B,D) at two different magnifications. According to the SEM micrograph in Figure 2A,B, TPS loaded with 20 wt. % KF shows interstices around KF and empty holes caused by fibre extraction. Different fibre sizes were observed, with lengths of around 40–100 µm and diameters of approximately 30–50 µm. A similar morphology was observed for TPS/20CF composite in Figure 2C,D, where spaces around CF and footprints of fibre extraction are apparent. Coconut fibres showed a width of around 40–85 µm. According to Figure 2, the morphology of TPS/20KF and TPS/20CF suggests that fibres act as fillers in TPS without any relevant chemical or physical interactions.

**Figure 3:**
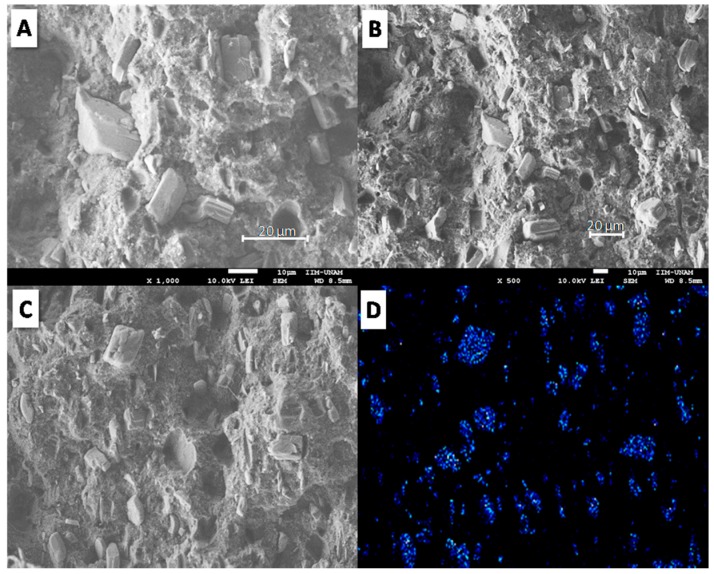
SEM micrographs of fractured surface of TPS/20APP at two different magnifications (**A**,**B**). Elemental analysis of TPS/20APP: (**C**) scanned area and (**D**) phosphorus mapping.

Figure 3 shows SEM micrographs of the fractured surface of TPS using 20 wt. % APP. According to Figure 3A,B, APP additive exhibits poor adhesion with TPS, characterized by empty holes and interstices between the particles and the polymer. However, APP particles showed good dispersion in the polymer matrix; even though large particle sizes (around 20 µm) were observed, no APP agglomerates were visible. This morphology was achieved by the double extrusion process to obtain the well-dispersed TPS composites. In order to confirm the presence and dispersion of APP particles on the fractured surface of TPS, phosphorus elemental analysis was carried out by energy dispersive spectroscopy (EDS) of TPS/20APP composite (Figure 3C,D). Good dispersion of the phosphorus is shown by EDS analysis of the TPS/20APP fractured surface.

**Figure 4:**
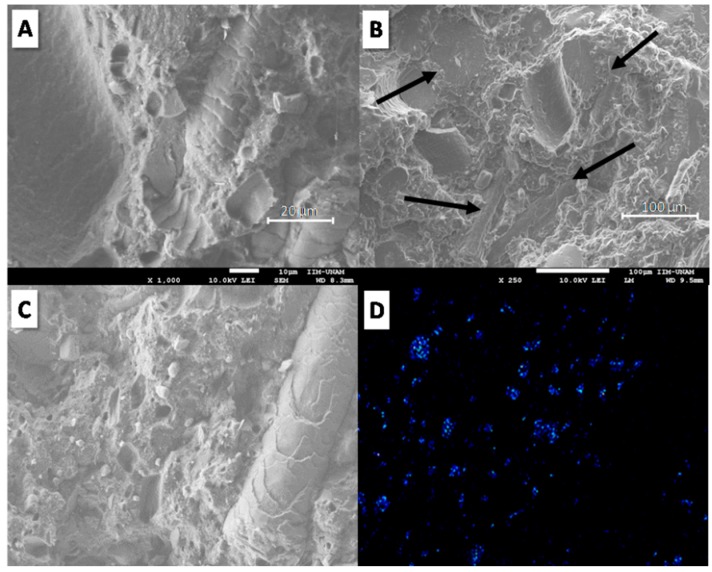
SEM micrographs of the fractured surface of TPS/20KF/10APP at two different magnifications (**A**,**B**). Elemental analysis of TPS/20KF/10APP: (**C**) scanned area and (**D**) phosphorus mapping.

**Figure 5:**
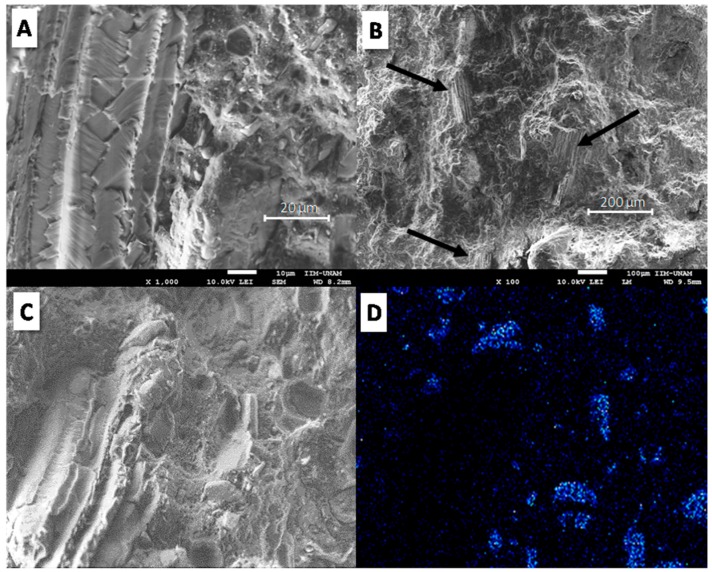
SEM micrographs of TPS/20CF/10APP at two different magnifications (**A**,**B**). Elemental analysis of TPS/20CF/10APP: (**C**) scanned area and (**D**) phosphorus mapping.

Figure 4 reveals the morphology of the fractured surface at different points and phosphorus elemental analysis of the TPS/20KF/10APP composite. According to Figure 4A,B, keratin fibres appear well embedded in the polymer matrix (indicated by black arrows); no interstices around the fibres were observed. With respect to APP particles, rather poor adhesion on the fractured surface was still apparent. Figure 4C,D shows the SEM image and the mapping of phosphorus in TPS/20KF/10APP; the micrographs reveal the presence and dispersion of APP particles. Figure 5 presents SEM micrographs and phosphorus elemental analysis of the fractured surfaces of the TPS/20CF/10APP composite. Figure 5A,B reveals dispersed coconut fibre and shows improved mechanical coupling around the fibre. Figure 5C,D illustrates the presence of APP particles with rather poor adhesion with the polymer matrix. According to morphological analyses of TPS/20KF/10APP and TPS/20CF/10APP composites (Figure 4 and Figure 5 respectively), fibres added in combination with APP appeared better embedded on the polymer matrix than the composites without APP additive, probably because the higher load (30 wt. %) produces less polymer contraction in the composites.

### 3.2. Mechanical Properties

Table 2 presents the mechanical properties of the investigated composites. According to Table 2, TPS shows ductile behaviour: a low Young’s modulus (128 MPa), along with high strain at break (463%), tenacity (89 MPa) and Izod impact resistance (no break), as expected since this kind of biopolymer has traditionally been used for packaging applications [28]. Regarding the effect of keratin fibre contents on the mechanical properties of TPS composites, Table 2 reports that increasing KF content in TPS causes a reduction in strain at break, tenacity and Izod impact resistance. As an example, when KF was added at 15 and 30 wt. % (TPS/15KF and TPS/30KF), the strain at break was reduced from 463% to 190% and 26%, tenacity from 89 MPa to 19 and 2 MPa, Izod impact resistance from no break to 397 and 81 J/m, respectively. The Young’s modulus increased from 128 MPa to 177 and 229 MPa, respectively. Tensile strength exhibited similar results for TPS/15KF and TPS/30KF composites, 11 and 10 MPa, respectively. Comparable mechanical behaviour was observed when CF was added to TPS: high CF content resulted in a high Young’s modulus along with low strain at break, tenacity and Izod impact resistance. Taking a comparison of TPS, TPS/10CF and TPS/30CF as an example, strain at break was reduced from 463% to 207 and 13%, tenacity from 89 MPa to 31 and 2 MPa, Izod impact resistance from no break to 389 and 81 J/m, each in that order. Tensile strength presented the same value, 18 MPa, for both composites (TPS/10CF and TPS/30CF). The Young’s modulus exhibited a remarkable increase, from 128 MPa to 168 and to 474 MPa, respectively, due to the high rigidity of coconut fibre [29]. According to the results on mechanical properties (Table 2), the increasing fibre content (KF and CF) in the TPS produced rigid and fragile materials, as already observed in natural fibre polymer composites [30]. A compatibilizer may be used in the future to obtain better mechanical coupling.

As is well known, the addition of high content of flame retardants to polymer matrix may have a significant effect on mechanical properties [31]. The addition of 20 and 30 wt. % APP to TPS increased brittleness, as seen in TPS/10APP and TPS/15APP. Similarly, the TPS/20APP composite showed lower tensile strength, strain at break, tenacity and Izod impact resistance than TPS. The Young’s modulus exhibited an increase from 128 to 163 MPa for TPS and TPS/20APP, respectively. The reinforcement achieved by APP is similar to that for CF.

Regarding KF in combination with the APP effect, Table 2 reports on TPS/15KF/15APP and TPS/20KF/10APP composites with different KF and APP contents but the same total load (30 wt. %), compounded in order to replace part of the APP flame retardant additive (5 or 10 wt. %) using KF. The resulting properties exhibited a similar Young’s modulus, tensile strength and tenacity. Nevertheless, strain at break and Izod impact resistance were somewhat dependent on the composition of TPS/KF/APP: TPS/15KF/15APP exhibited 54% strain at break and 118 J/m Izod impact resistance, while TPS/20KF/10APP showed 45% strain at break and 128 J/m Izod impact resistance. Regarding CF in combination with APP (TPS/10CF/15APP and TPS/20CF/10APP composites), the mechanical properties were influenced by the CF content, as was observed in TPS/CF composites; high CF content reduced strain at break, tenacity and Izod impact resistance, while the Young’s modulus was increased. 

Nevertheless, although high KF and CF content, namely 30 wt. %, produces deteriorated mechanical properties, when KF and CF fibres were added at 20 wt. % in combination with 10 wt. % APP (30 wt. % total load), the corresponding mechanical properties were slightly improved over those achieved by adding only KF or CF at 30 wt. % (TPS/30KF and TPS/30CF). The combination of 20 wt. % KF and 10 wt. % APP (TPS/20KF/10APP) produced a less fragile and more ductile material than TPS/30KF. The Young’s modulus, tensile strength, strain at break, tenacity and Izod impact resistance increased from 229 to 243 MPa, 10 to 13 MPa, 26 to 45%, 2 to 5 MPa and 81 to 128 J/m, respectively. This finding was similar to the combination of 20 wt. % CF with 10 wt. % APP (TPS/20CF/10APP) as compared to the TPS/30CF composite; strain at break and Izod impact resistance were slightly improved. In this context, the mechanical improvement in TPS/20KF/10APP and TPS/20CF/10APP composites over TPS/30KF and TPS/30CF indicates better stress transfer due to some physical fibre-polymer interactions [32], as was observed in morphological analyses (the fibres appeared well embedded in the polymer matrix, Figure 4 and Figure 5).

### 3.3. Rheology

**Figure 6:**
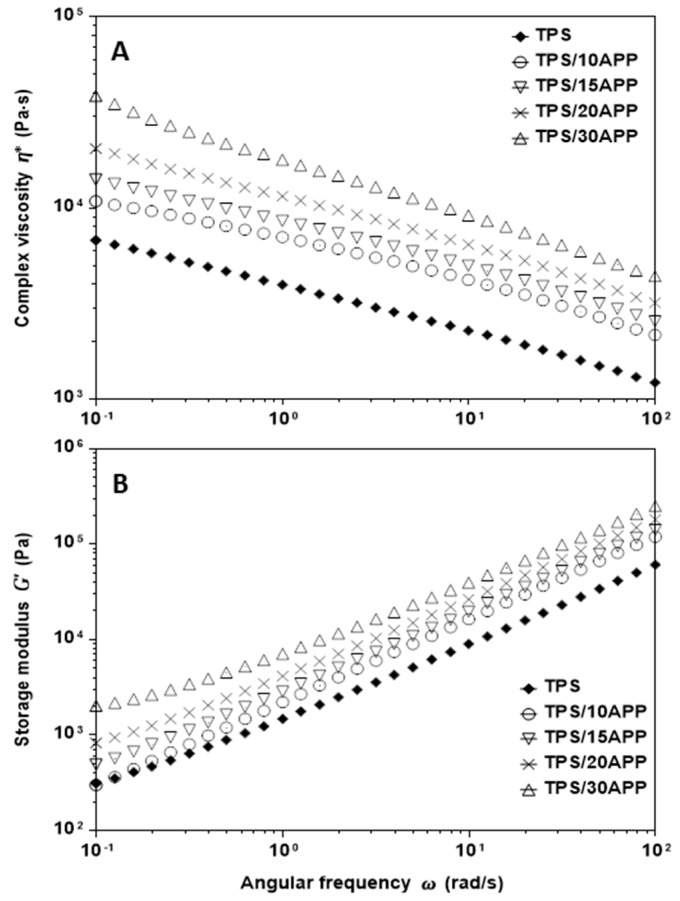
Complex viscosity (**A**) and storage modulus (**B**) as a function of the oscillatory frequency of TPS and TPS/APP composites with varied contents.

The rheological properties of TPS composites were assessed by oscillatory tests under continuous simple and small amplitude oscillatory shear flow (SAOS). Figure 6 displays complex viscosity (Figure 6A) and storage modulus (Figure 6B) as a function of angular frequency for TPS with different contents of 10, 15, 20 and 30 wt. % APP (TPS, TPS/10APP, TPS/15APP, TPS/20APP and TPS/30APP). According to the data plotted in Figure 6A, the complex viscosity of TPS and TPS/APP composites exhibited remarkable dependency on angular frequency, with complex viscosity decreasing as angular frequency increased. This behaviour is similar to the shear thinning effect observed in shear flow tests (not shown in this work). On the other hand, high APP content (20 and 30 wt. %) exerted an influence on complex viscosity throughout the entire frequency range (0.1–100 rad/s) greater than that for TPS. Regarding elastic properties, Figure 6B shows the storage modulus as a function of the angular frequency of TPS and TPS/APP composites (at 10, 15, 20 and 30 wt. %): the storage modulus exhibited higher values for samples using 20 and 30 wt. % APP than for TPS over the entire frequency range. The highest content of APP (TPS/30APP) produced the most elastic material, observed in the low frequency range (0.1–1.0 rad/s).

**Figure 7:**
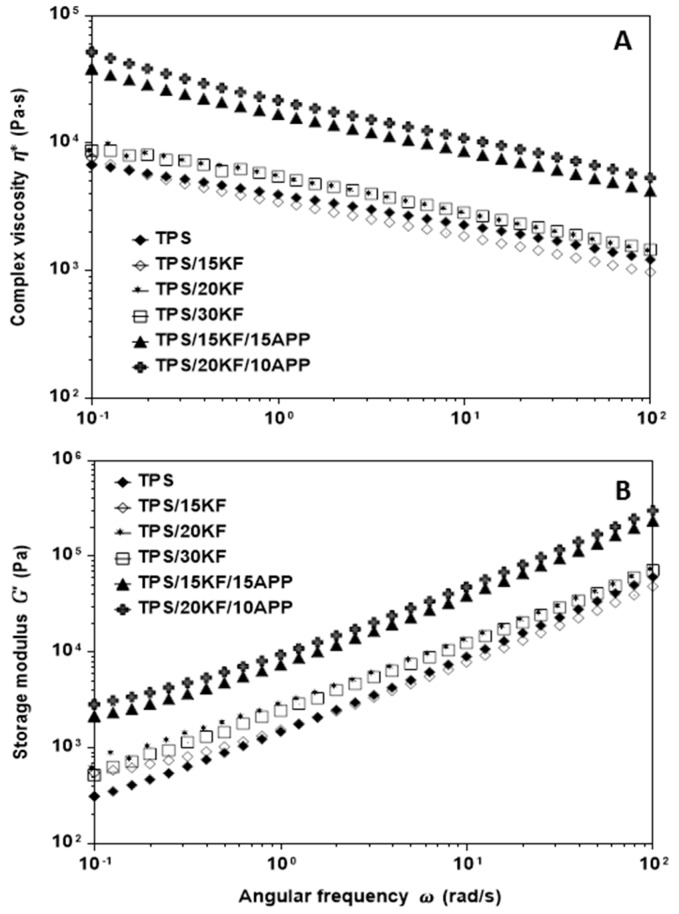
Complex viscosity (**A**) and storage modulus (**B**) as a function of the oscillatory frequency of TPS, TPS/KF and TPS/KF/APP composites with varied content.

Figure 7 presents the effect of KF content and KF/APP combination on flow behaviour; complex viscosity (Figure 7A) and storage modulus (Figure 7B) are plotted as a function of angular frequency. Regarding the effect of KF content, Figure 7A shows that TPS/KF composites exhibited a rheological pattern similar to TPS; in the low frequency range (0.01–0.1 rad/s) the TPS/15KF composite presented curves overlapping with TPS, whereas in the high frequency range (1.0–100 rad/s), complex viscosity was slightly lower than for TPS. Meanwhile, TPS/20KF and TPS/30KF viscosity was slightly higher than TPS over the entire frequency range. In general, for all TPS/KF composites as well as TPS, complex viscosity showed a dependency on angular frequency similar to shear thinning behaviour in simple shear flow. The shear thinning effect was observed for all TPS/KF composites regardless of KF content. In this regard, it is reasonable to think that keratin fibres are well oriented under flow, which is consistent with similar natural fibre composites [33,34]. Figure 7B reveals that TPS/15KF and TPS have a similar storage modulus, with the TPS/15KF and TPS curves overlapping in the 0.1–1.0 rad/s range, while the storage modulus values for TPS/20KF and TPS/30KF were slightly higher than TPS over the entire frequency range. It is interesting to observe that the rheological behaviour of composites using keratin fibres depended mainly on the composition of the materials. Figure 7 shows that the KF and APP combination has a remarkably stronger effect on the complex viscosity and storage modulus than KF alone. When KF and APP were added to TPS (TPS/15KF/15APP and TPS/20KF/10APP), complex viscosity and storage modulus increased over those of TPS and TPS/KF composites throughout the entire frequency range. However, rheological analyses point out that when KF are added in combination with APP, processing becomes more difficult than for KF without APP.

**Figure 8:**
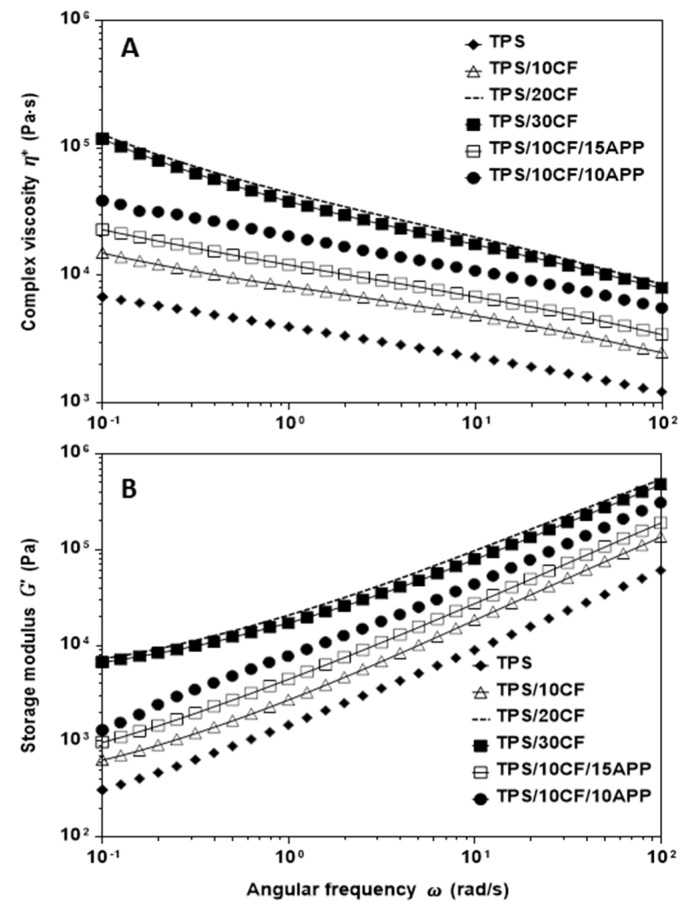
Complex viscosity (**A**) and storage modulus (**B**) as a function of the oscillatory frequency of TPS, TPS/CF and TPS/CF/APP composites with varied content.

Figure 8 displays the rheological behaviour of TPS using different contents of CF and the CF/APP combination. As it was observed for TPS, TPS/APP and TPS/KF composites, all composites using coconut fibre (Figure 8A) showed shear thinning behaviour (complex viscosity was reduced as frequency increased). On the other hand, CF content exhibited an important effect on complex viscosity; high CF content (20 and 30 wt. %) produced much higher complex viscosity than TPS, TPS/10CF, TPS/10CF/15APP and TPS/20CF/10APP, suggesting that CF behaved as a filler without physical or chemical interaction with the polymer matrix. Regarding storage modulus, Figure 8B shows an increase for TPS/10CF, TPS/10CF/15APP and TPS/20CF/10APP composites with reference to TPS but a storage modulus pattern similar to TPS (similar slopes). TPS/20CF and TPS/30CF composites exhibited much higher elastic properties than TPS/10CF, TPS/10CF/15APP and TPS/20CF/10APP composites. In this regard, it is possible to point out that the combination of coconut fibres with APP appears to favour the processing of these materials.

### 3.4. Thermal Decomposition

Thermal decomposition under nitrogen was investigated using thermogravimetric analysis. TPS as well as the formulations with CF, KF, APP and their combinations were analysed to make a statement about altered decomposition pathways. Results of the thermogravimetry are displayed in Table 3. The temperature at 5 wt. % mass loss (Tat 5% Mass Loss) characterizes the beginning of the decomposition, the temperature at the maximum mass loss rates (Tmax) and the mass loss step (Δmass) the decomposition steps.

**Figure 9:**
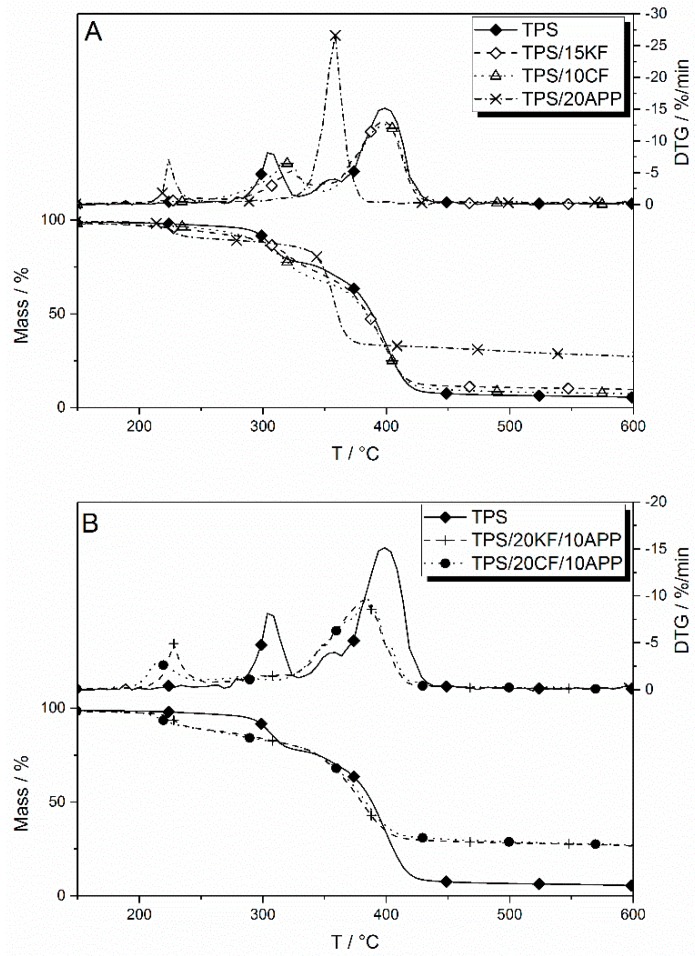
Mass and mass loss rates (DTG) of TPS blended with single natural fibre components or APP (**A**) and of combinations of TPS with natural fibres and APP (**B**).

TPS shows two main decomposition steps, which are clearly visible in the mass loss rate (DTG) curve (Figure 9A). The first peak is attributed to the starch phase, with occurred at a temperature of 304 °C. The second decomposition step, at 402 °C, is associated with the polyester phase of the TPS. In the DTG, a shoulder is visible prior to the second peak, which derived from the additional additives incorporated in the commercial blend [35].

Both fibres, KF and CF, increased the residue in thermogravimetry in a similar manner with increasing fibre content. The decomposition temperature of the first decomposition step was shifted towards higher temperatures, by 18 °C for 15 wt. % KF and by 22 °C for 10 wt. % CF in TPS, while the temperature for the second decomposition step remained more or less constant. The incorporation of natural fibres led to slightly reduced mass loss in both decomposition steps.

When APP was present in TPS formulations, the pyrolysis and decomposition temperatures changed significantly. With 20 wt. % of APP in TPS, the first decomposition step occurred at around 225 °C and was associated with the release of ammonia. A second decomposition step occurred at 359 °C, a temperature between the first and second steps of the reference TPS. This indicates an interaction between the TPS and the APP additive that led to an altered decomposition pathway during pyrolysis. The amount of residue formed by TPS/20APP is greatly increased, to 19 wt. % more than TPS.

Figure 9B presents mass and mass loss rates (DTG) curves for combinations of natural fibres and APP. They show the same first decomposition step as TPS/20APP, which is attributed to the loss of ammonia. This decomposition step occurred at similar temperatures when KF was incorporated into the APP-TPS blend and was shifted to lower temperatures when CF was used as a natural fibre filler. The second decomposition step was altered as compared to only APP in TPS, in a similar fashion for both fibre fillers. The maximum temperature of the second decomposition step was shifted to temperatures around 20 °C higher. Decomposition also happened at a slower pace and over a broader temperature range compared to only APP in TPS, as seen in a lower but broader peak in the DTG curves. This indicates an interaction between the fibre fillers and APP during thermal degradation. The combination of natural fibres and APP also led to increased residue formation. When comparing the additional residue of TPS/15KF/15APP, the measured residue of 27 wt. % was slightly more than the expected residue of 25 wt. %, suggesting slightly synergistic behaviour, while the formulation TPS/20KF/10APP achieved superposition in residue formation. The expected residue formation of around 24 wt. % for the combination TPS/10CF/15APP was similar to the measured residue amount of 25 wt. %. A good synergistic effect in residue formation is observed in TPS/20CF/10APP, with 24 wt. % compared to an expected amount of only 21 wt. %.

### 3.5. Flammability

The flammability of TPS biocomposites with the natural fibre fillers KF and CF, APP and the combination of both, were characterized by determining their limiting oxygen index and their classification in the UL 94 vertical test. UL 94 vertical test evaluates the reaction of the plastic materials to a small flame. The classification of this method depends on the flammability characteristics. For example, V0 classification means that the burning stops within 10 s for each individual specimen, after the first or second flame application to the specimen. No flaming drips are allowed. Meanwhile, V2 rating means that the burning stops within 30 s for each individual specimen, after the first or second flame application to the specimen. Flaming drips are allowed. The results of both flammability tests are summarized in Table 4.

TPS as well as TPS-natural fibre biocomposites did not achieve a rating in the UL 94 vertical test. With increasing fibre content, the OI increased only slightly. However, a loading of 20 wt. % of APP in TPS resulted in an OI of 29 vol. % and was also the lowest concentration measured to achieve a V-0 classification in the UL 94 vertical test. It is remarkable that the minimum APP content to achieve a rating in the vertical test was lowered by the addition of keratin or coconut fibres. Even with 20 wt. % fibre content and only 10 wt. % APP content, a V-2 rating was achieved. OI values for all combinations of fibres and APP were in the range of 27 to 29 vol. %.

**Figure 10:**
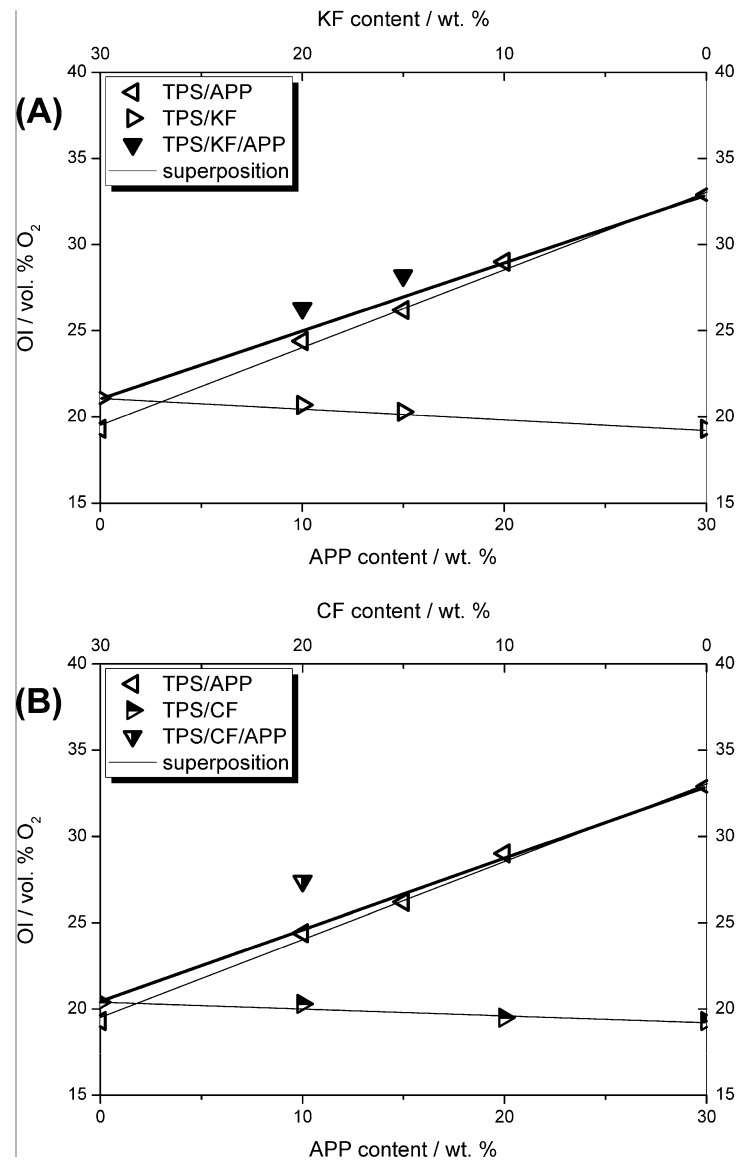
Content dependency and synergy of oxygen index for KF (**A**) and CF (**B**) formulations. Thin lines represent the linear behaviour of the OI for the single components derived from OI measurements of TPS/APP (hollow triangles pointing left), TPS/KF (hollow triangles pointing right) and TPS/CF (half-filled triangles pointing right) formulations, the thick line represents the calculated superposition of the fibre/APP combinations. The OI measured for fibre/APP combinations in TPS are displayed as solid triangles pointing down (TPS/KF/APP) and half-filled triangles pointing down (TPS/CF/APP).

Figure 10 displays the change in OI behaviour with increasing additive contents, that is, fibre, APP and combinations of natural fibres and APP. In the graphs, the thin solid lines represent the linear dependency of the OI on the additive content of a respective single component in TPS, while the thick solid line shows the oxygen index behaviour if a combination of both additives, fibres and APP, were to result in a superposition. For the combinations of fibres and APP that amount to an overall additive content of 30 wt. %, namely TPS/15KF/15APP, TPS/20KF/10APP (Figure 10A) and TPS/20CF/10APP (Figure 10B), the measured oxygen indices are all located above the superposition line. This illustrates synergistic behaviour in OI. For CF, this synergism between fibres and APP was even more pronounced than for formulations with KF and APP, suggesting a better interaction between CF and APP during burning in terms of flame retardancy. These synergistic effects also explain the improved UL 94 rating for natural fibre-APP combinations.

The synergistic effect index was determined by calculating the theoretical superposition for linear behaviour using Equation (1) [36,37]. The OI for TPS/20CF and TPS/10APP increased compared to TPS by 1 vol. % and 5.1 vol. %, respectively. The calculated superpositioned OI increase for the formulation containing 20 wt. % of CF and 10 wt. % amounted to 6.1 vol. %. The oxygen index increase for TPS/20CF/10APP was measured to be 7.6 vol. %. Comparing the calculated superposition and the measured oxygen index increase yielded a synergistic effect index of 1.25.

(1) $${\mathrm{SE}}_{\mathrm{abs}}{\left(\mathrm{OI}\right)}_{x,y=\mathrm{const}.}=\frac{\Delta \mathrm{OI}\left(\mathrm{TPS}/20\mathrm{CF}/10\mathrm{APP}\right)}{\Delta \mathrm{OI}\left(\mathrm{TPS}/20\mathrm{CF}\right)+\Delta \mathrm{OI}\left(\mathrm{TPS}/10\mathrm{APP}\right)}=\frac{7.6\;\mathrm{vol}.\;\%}{1\;\mathrm{vol}.\;\%+5.1\;\mathrm{vol}.\;\%}=1.25.$$

For TPS/20KF/10APP, the synergistic effect index, calculated in the same manner, amounted to 1.08. Through thorough observation of the OI flammability measurements, the synergistic effect, especially between CF and APP, is attributed to enhanced effectiveness in the condensed phase. This synergistic behaviour would also be expected in forced flaming combustion tests conducted in the cone calorimeter.

### 3.6. Burning Behaviour under Forced Flaming Combustion

**Figure 11:**
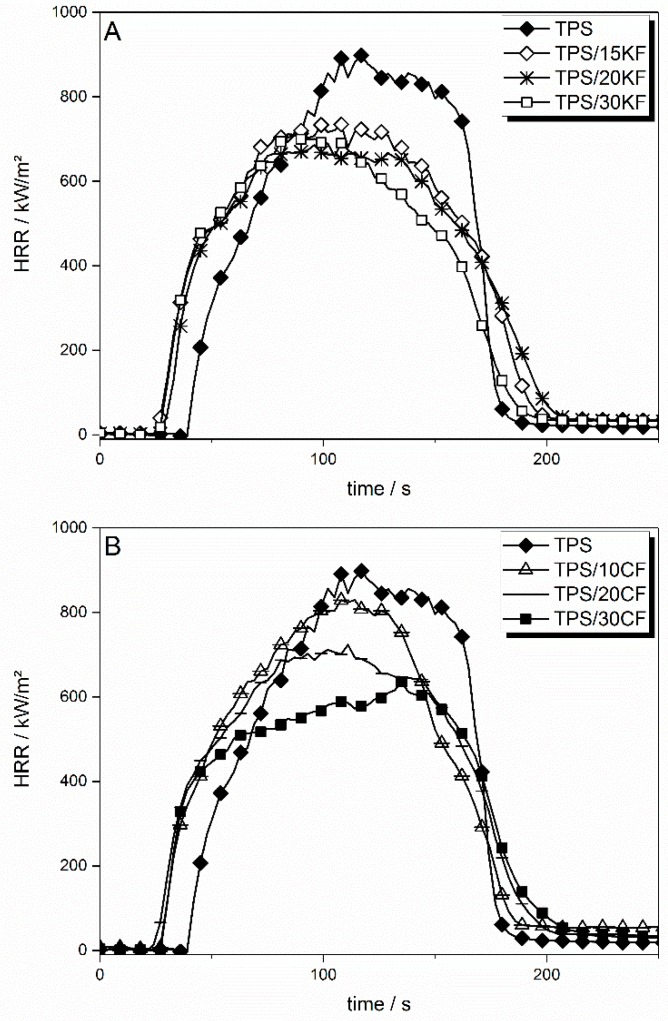
HRR curves for TPS reinforced with different amounts of keratin (**A**) and coconut fibres (**B**) in comparison to neat TPS.

The flame retarding effect of only keratin or coconut fibres in TPS is mainly limited to a PHRR (peak heat release rate) reduction of up to 33% and a THE (total heat evolved) reduction of up to 11%. Cone calorimeter results are shown in Table 5. The heat release rate (HRR) curves of natural fibres in TPS are shown in Figure 11.

The incorporation of the natural fibres KF and CF had various effects on the HRR of TPS. For both sorts of fibres, the time to ignition (tig) was shifted to lower temperatures, from 37 s to around 25 s for 30 wt. % of fibres, respectively. Increased viscosity due to the addition of fibres resulted in later liquefaction of the sample surface in the cone calorimeter, reducing the heat exchange and thus cooling via convection. As a consequence, the sample heated up more quickly and ignited earlier. KF (Figure 11A) at 15 wt. % reduced the PHRR by around 200 kW/m^2^. With higher amounts of KF, the reduction in PHRR levelled off. Apart from a slightly slower HRR decay after flameout, the overall burning behaviour was similar to non-flame retarded TPS. The effective heat of combustion, displayed here as total heat evolved (THE) divided by total mass loss (TML), was not influenced by the KF incorporated in TPS. They had an influence neither on the combustion efficiency of released fuel nor on the heat of combustion of the volatiles. When CF was incorporated into TPS, the levelling off of the reduction in PHRR was less pronounced, resulting in a lower PHRR for TPS/30CF (Figure 11B). The burning behaviour of TPS displayed a more characteristic change in the HRR curve with increasing amounts of CF than with added KF. For TPS/30CF, the PHRR was shifted to later times, just before flameout (tfo) and the fire growth behaviour in the beginning was reduced. This resulted in a decrease in the slope of the HRR curve after the initial rise. This indicates a more pronounced protective layer becoming visible in the HRR curve shape. THE/TML was only insignificantly decreased through the addition of CF. CF also produced a higher amount of residue, with 12.4 wt. % at a loading of 30 wt. % compared to 10.7 wt. % for TPS/30KF, hinting at higher thermal stability of the coconut fibres. In general, residue formation in the cone calorimeter was similar to thermogravimetric residue observation. 

**Figure 12:**
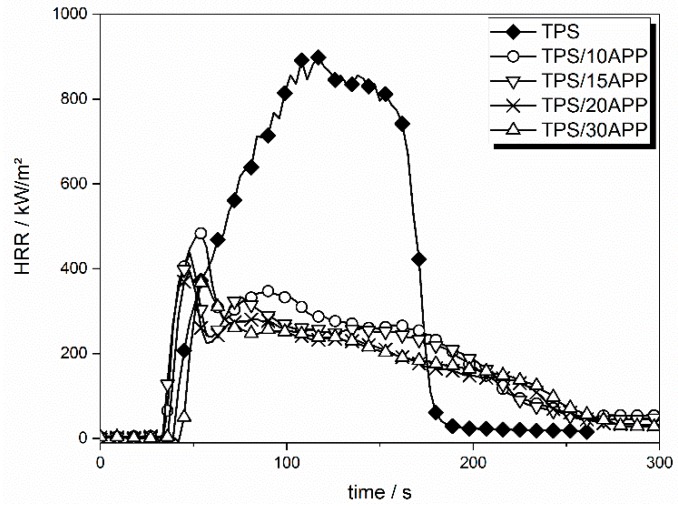
HRR curves of TPS formulations with APP.

The incorporation of APP in TPS resulted in a clear change in HRR curve shape and thus in burning behaviour, as compared to that of a charring material [38]. The HRR curves of 10, 15, 20 and 30 wt. % APP are displayed in Figure 12.

HRR changed significantly for formulations with APP as compared to TPS. The initial peak in HRR after ignition, the ensuing local minimum and the slow decay of HRR, point to typical burning behaviour of material forming a protective layer. PHRR was decreased by around 50% with an APP load of just 10 wt. %. A loading of 20 wt. % APP lowered the PHRR by an additional 9%. Even higher APP loadings no longer significantly reduced the PHRR, indicating that the effectiveness of APP levels off. A similar trend is seen in the reduction of fire load (total heat evolved, THE). The THE was reduced by 42% at an APP loading of 10 wt. %. The highest APP load in TPS, which was 30 wt. %, resulted in a THE reduction of 52%. The addition of APP to TPS induced char formation, yielding 23 wt. % of residue at an APP load of 10 wt. %. A levelling off trend was also observed in residue formation with increasing APP load. Considering the initial residue amount of 4 wt. % for TPS, the addition of 10 wt. % APP yielded an additional residue formation of 19 wt. %. Incorporation of 30 wt. % APP in TPS resulted in an additional residue amount of 31 wt. %. APP also led to a reduction of up to 30% in THE/TML. Otherwise released carbonaceous species which act as fuel for the flame were stored in the formed residue, reducing the heat of combustion. Additionally, APP released NH_3_, which does not contribute to the heat production, diluting the flame and therefore reducing combustion efficiency.

**Figure 13:**
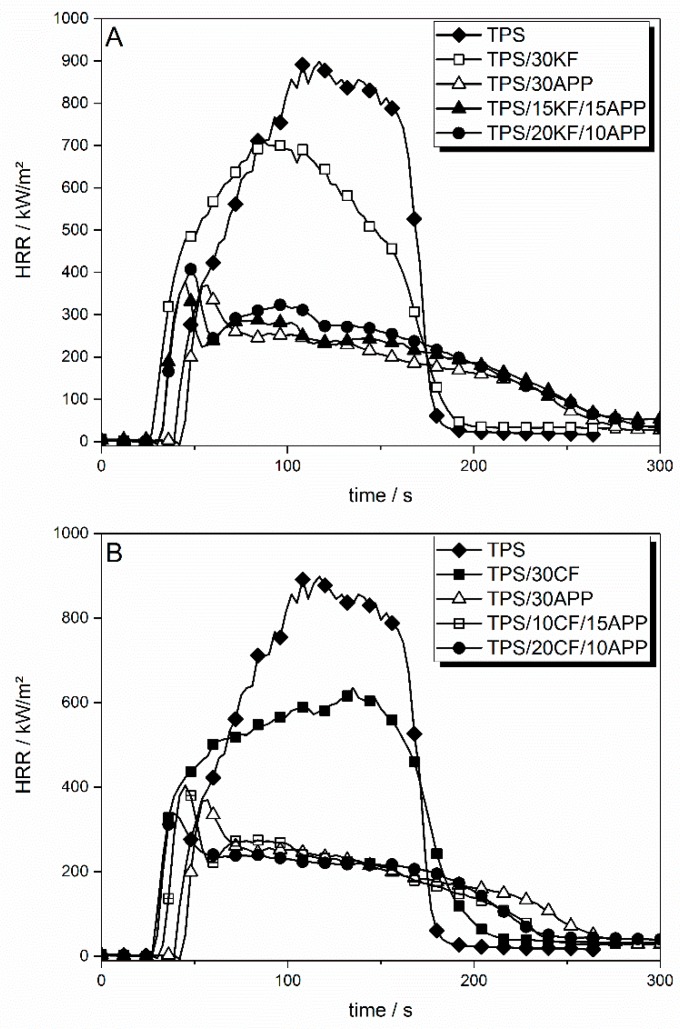
HRR curves for combinations of APP with keratin fibres (**A**) and coconut fibres (**B**).

Natural fibre reinforced TPS in combination with APP yielded similar HRR results as TPS formulations with only APP as a flame retardant additive. However, it is possible to replace certain amounts of the flame retardant APP with fibre content to achieve similarly good results as with 20 or 30 wt. % of APP alone. Combinations of natural fibre filler and APP are shown in Figure 13.

Figure 13A displays the HRR curves derived from cone calorimeter measurements for a total additive amount of 30 wt. % in TPS. The KF-APP combinations TPS/15KF/15APP and TPS/20KF/10APP showed similar HRR results as TPS/30APP. TPS/15KF/15APP exhibited a reduction in PHRR to 379 kW/m^2^ and a reduction in THE to 49.9 MJ/m^2^, while TPS/20KF/10APP reduced the PHRR to 407 kW/m^2^ and the THE to 54.3 MJ/m^2^. The HRR was dominated by the addition of APP. The nonlinear levelling off of effectiveness with increasing APP content enabled strong synergy.

Combinations of CF and APP in TPS showed slightly different behaviour (Figure 13B). While the PHRR of TPS/10CF/15APP was still a bit higher than the PHRR of TPS/30APP, at 407 kW/m^2^ compared to 376 kW/m^2^, the THE was reduced to 42.2 MJ/m^2^ as opposed to 43.4 MJ/m^2^. A comparison of TPS/20CF/10APP and TPS/30APP shows that the overall flame retardancy performance may be even better even though a total additive load of 30 wt. % is maintained. The addition of 20 wt. % CF to the mixture of 10 wt. % APP in TPS resulted in a decrease in PHRR, which was not possible by increasing APP content alone. In terms of fire load, the THE of TPS/30APP and TPS/20CF/10APP were similarly low, at around 43.3 to 43.1 MJ/m^2^.

For CF, a synergistic effect was observed in both PHRR and THE. In order to obtain the synergistic effect index as a statement for the significance of the synergism, Equation (2) is used [34], with M being the result derived from cone calorimeter measurement. This approach was chosen over the proportionate approach, in which the total additive load is kept constant, because there was no significant reduction in cone calorimeter results for TPS with 10, 20 or 30 wt. % APP. Since cone calorimeter results like PHRR or THE did not decrease in a linear fashion, the relative synergistic effect index was calculated as a more correct concept.

(2) $${\mathrm{SE}}_{\mathrm{rel}}{\left(M\right)}_{x,y=\mathrm{const}.}=\frac{1-\Delta M\left(\mathrm{TPS}/20\mathrm{CF}/10\mathrm{APP}\right)}{1-\Delta M\left(\mathrm{TPS}/20\mathrm{CF}\right)\times \Delta M\left(\mathrm{TPS}/10\mathrm{APP}\right)}.$$

In TPS/20CF, the PHRR was reduced to 727 kW/m^2^. APP at a load of 10 wt. % reduced the PHRR of TPS by around 50%. This resulted in an expected PHRR of TPS/20CF/10APP of around 366 kW/m^2^, assuming no synergistic effect. However, the measured PHRR of TPS/20CF/10APP was 338 kW/m^2^, yielding a synergistic effect index of 1.08. For THE, the synergism between CF and APP became even clearer. The calculated superposition in THE for TPS/20CF/10APP amounted to 50.9 MJ/m^2^ and the measured THE was 43.1 MJ/m^2^. Using Equation (2), this yielded a synergistic effect index of 1.2.

**Figure 14:**
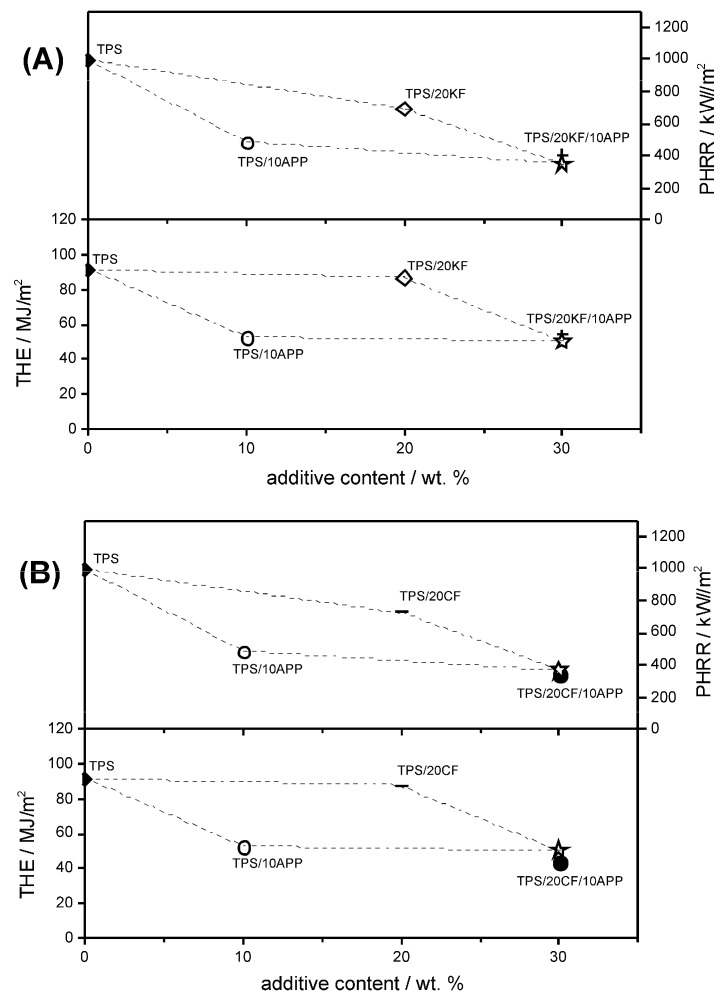
Visualization of superposition (hollow star) and measured PHRR and THE of TPS/20KF/10APP (**A**) and TPS/20CF/10APP (**B**).

The effect of combining fibres and APP is visualized in Figure 14. The trapezoid schematics are constructed to illustrate the superposition values for 20 wt. % natural fibre and 10 wt. % APP in TPS. Hollow stars mark the superposition values of THE and PHRR for TPS/20KF/10APP (Figure 14A) and TPS/20CF/10APP (Figure 14B). For TPS/20KF/10APP, the measured values for THE and PHRR are located around the hollow star marking the calculated superposition. This illustrates the occurrence of a superposition effect of this combination. On the other hand, TPS/20CF/10APP showed clear synergistic behaviour in PHRR and especially in THE, since the measured values are located below the hollow star superposition.

Since APP and the natural fibre fillers showed their activity as flame retarding additives mostly in the condensed phase, the reason for the synergistic effect of CF in combination with APP is found by analysing the cone calorimeter residues.

**Figure 15:**
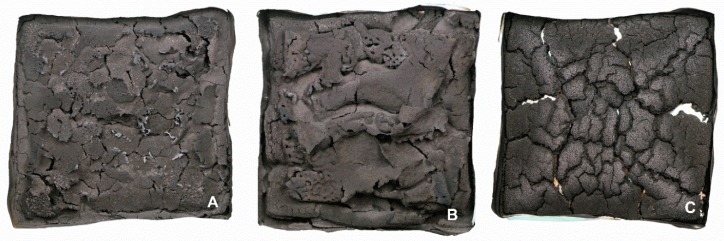
Cone calorimeter residue photographs of TPS/10APP (**A**), TPS/20KF/10APP (**B**) and TPS/20CF/10APP (**C**).

Figure 15 shows the character of the cone calorimeter residues of TPS/10APP (Figure 15A) and the two fibre-APP combinations TPS/20KF/10APP (Figure 15B) and TPS/20CF/10APP (Figure 15C). When 10 wt. % of APP were incorporated in the TPS matrix, the formed residue was very light and brittle. The relatively smooth surface was disrupted by small cracks, resulting in a flaky and flocculent residue. Adding 20 wt. % of KF resulted in a more continuous surface but the overall character and nature of the residue was very similar to that of TPS/10APP. When 20 wt. % of CF were combined with 10 wt. % of APP, the char structure of the residue changed completely. Instead of a light and brittle char, the residue was very stable and showed a resemblance to wood char, pervaded by relatively large cracks. The change in char structure becomes clearer in the SEM micrographs of the respective residues.

**Figure 16:**
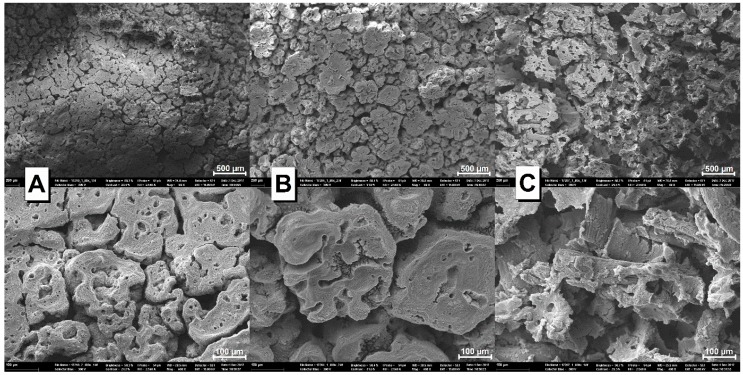
SEM micrographs of the cone calorimeter residue surfaces of TPS/10APP (**A**), TPS/20KF/10APP (**B**) and TPS/20CF/10APP (**C**) at two different magnifications (top and bottom).

Comparing the surfaces of TPS/20KF/10APP (Figure 16B) and TPS/10APP (Figure 16A) in SEM makes the similarities much clearer. Almost no differences are observed. It must be noted that no residual KF were found on the surface of the investigated TPS/20KF/10APP residue, so they do not contribute to the residue structure. In contrast to this, the residual CF were clearly visible in the TPS/20CF/10APP (Figure 16C) residue. CF reinforced the APP-induced char, resulting in a completely different char structure. This becomes even clearer at 400x magnification, where interconnection and coating of the CF with residue is apparent. The synergistic effect of CF in combination with APP in TPS is therefore attributed to the physical enhancement of the residue in TPS/20CF/10APP.

## 4. Conclusions

With respect to mechanical properties, the addition of KF and CF to TPS has been demonstrated to produce rigid materials, as the Young’s modulus was increased. However, tensile strength, strain at break, tenacity and Izod impact resistance were reduced. Comparing the effects of keratin and coconut fibre, CF produces a more rigid material than KF, namely at high content, 30 wt. %. On the other hand, the combination of KF or CF with APP (TPS/20KF/10APP or TPS/20CF/10APP) produces more ductile materials than those using 30 wt. % of KF or CF. 

Regarding rheological properties, the addition of KF to TPS did not significantly affect the flow properties of TPS. However, when keratin fibres were added in combination with APP, complex viscosity increased, making processing more difficult. By contrast, when CF were added in combination with APP, the flow properties benefited and thus the processing of the composites also improves as compared with materials containing KF or CF without the flame retardant APP.

The flammability behaviour of TPS was greatly improved through the addition of APP. This was confirmed by both UL 94 and OI tests. In terms of the oxygen index, a synergistic effect was ascertained between APP and both fibres, KF and CF. For 20 wt. % KF and 10 wt. % APP in TPS, a synergistic effect index of 1.08 was calculated, while 20 wt. % CF in combination with 10 wt. % APP resulted in a synergistic effect index of 1.25. This indicated that CF improved the flame retardancy effectiveness of APP in TPS.

Investigating the fire behaviour under forced flaming conditions in the cone calorimeter revealed a change toward charring burning behaviour when the flame retardant APP was added. While the combination of KF and APP showed a good superposition, CF was able to further reduce PHRR and THE to values lower than those with APP as a single flame retardant additive. The synergistic effect of CF in combination with APP was quantified to an index of 1.08 in PHRR and 1.2 in THE reduction. This synergistic effect was attributed to the residue structure, since CF was able to reinforce the formed char, increasing residue stability. This was confirmed with SEM micrographs.

All in all, the investigation and characterization of the natural fibre fillers KF and CF for TPS enabled conclusions about mechanical and processing properties as well as great insights into flame retardancy behaviour. The combination of APP and industrial waste fibres is proposed as an interesting route towards sustainable flame retarded materials.

## Figures and Tables

**Table 1:** Composition of investigated TPS-based biocomposites.

Composition	Acronym
Mater-Bi EF05B	TPS
TPS + 15% keratin fibres	TPS/15KF
TPS + 20% KF	TPS/20KF
TPS + 30% KF	TPS/30KF
TPS + 10% coconut fibres	TPS/10CF
TPS + 20% CF	TPS/20CF
TPS + 30% CF	TPS/30CF
TPS + 10% ammonium polyphosphate	TPS/10APP
TPS + 15% APP	TPS/15APP
TPS + 20% APP	TPS/20APP
TPS + 30% APP	TPS/30APP
TPS + 15% KF + 15% APP	TPS/15KF/15APP
TPS + 20% KF + 10% APP	TPS/20KF/10APP
TPS + 10% CF + 15% APP	TPS/10CF/15APP
TPS + 20% CF + 10% APP	TPS/20CF/10APP

**Table 2:** Mechanical properties of composites.

Acronym	Young’s Modulus ± σ (MPa)	Tensile Strength ± σ (MPa)	Strain at Break ± σ (%)	Tenacity ± σ (MPa)	Izod Impact Resistance ± σ (J/m)
TPS	128 ± 6	23 ± 1	463 ± 16	89 ± 8	non-break
TPS/15KF	177 ± 10	11 ± 0.3	190 ± 16	19 ± 2	397 ± 32
TPS/20KF	165 ± 13	11 ± 0.4	137 ± 23	13 ± 3	137 ± 0
TPS/30KF	229 ± 17	10 ± 0.1	26 ± 10	2 ± 1	81 ± 14
TPS/10CF	168 ± 6	18 ± 1	207 ± 4	31 ± 2	389 ± 26
TPS/20CF	322 ± 8	16 ± 1	40 ± 4	5 ± 1	230 ± 22
TPS/30CF	474 ± 25	18 ± 1	13 ± 2	2 ± 0.3	81 ± 20
TPS/10APP	124 ± 7	18 ± 0.5	368 ± 9	57 ± 2	non-break
TPS/15APP	138 ± 6	18 ± 0.3	336 ± 12	50 ± 2	non-break
TPS/20APP	163 ± 8	17 ± 0.3	233 ± 11	34 ± 4	213 ± 30
TPS/30APP	288 ± 11	15 ± 0.3	176 ± 7	24 ± 1	142 ± 21
TPS/15KF/15APP	247 ± 5	12 ± 1	54 ± 7	6 ± 1	118 ± 8
TPS/20KF/10APP	243 ± 3	13 ± 0.1	45 ± 3	5 ± 0.4	128 ± 8
TPS/10CF/15APP	225 ± 2	13 ± 0.1	61 ± 3	8 ± 0.1	147 ± 8
TPS/20CF/10APP	387 ± 5	14 ± 0.3	19 ± 5	2 ± 1	95 ± 14

**Table 3:** Thermogravimetry results for all formulations.

Materials	Tat 5% Mass Loss (°C)	Tmax1 (°C)	Tmax2 (°C)	Δmass1 (%)	Δmass2 (%)	Residue at 800 °C (wt. %)
TPS	288	304	402	22	74	4
TPS/15KF	238	322	397	25	67	7
TPS/20KF	250	326	395	27	62	9
TPS/30KF	244	325	390	31	54	13
TPS/10CF	258	312	399	31	61	6
TPS/20CF	250	313	398	33	58	7
TPS/30CF	260	313	397	36	50	13
TPS/10APP	229	230	370	11	70	18
TPS/15APP	228	227	358	10	66	22
TPS/20APP	217	225	359	9	68	23
TPS/30APP	225	225	363	9	70	20
TPS/15KF/15APP	211	223	361	9	63	27
TPS/20KF/10APP	214	229	383	11	66	22
TPS/10CF/15APP	213	220	360	10	64	25
TPS/20CF/10APP	206	215	384	10	64	24

**Table 4:** OI and UL 94 vertical test results of investigated biocomposites; no vertical rating (n. r.).

Formulation	OI (vol. %)	UL 94 Vertical
TPS	19.3	n. r.
TPS/15KF	20.3	n. r.
TPS/20KF	20.7	n. r.
TPS/30KF	21.1	n. r.
TPS/10CF	19.5	n. r.
TPS/20CF	20.3	n. r.
TPS/30CF	20.4	n. r.
TPS/10APP	24.4	n. r.
TPS/15APP	26.2	V-2
TPS/20APP	29.0	V-0
TPS/30APP	32.9	V-0
TPS/15KF/15APP	28.2	V-0
TPS/20KF/10APP	26.3	V-2
TPS/10CF/15APP	27.4	V-2
TPS/20CF/10APP	26.9	V-2

**Table 5:** Cone calorimeter results of investigated TPS biocomposites.

Acronym	tig (s)	tfo (s)	PHRR (kW/m^2^)	THE (MJ/m^2^)	TML (g)	Residue (%)	THE/TML (MJ/gm^2^)
TPS	37 ± 2	177 ± 3	960 ± 62	90.8 ± 0.7	44.0 ± 0.1	4.0 ± 0.0	2.1 ± 0.0
TPS/15KF	24 ± 2	193 ± 3	762 ± 1	90.1 ± 0.6	43.3 ± 0.2	8.0 ± 0.1	2.1 ± 0.0
TPS/20KF	28 ± 2	202 ± 7	690 ± 15	86.5 ± 4.2	42.1 ± 1.9	9.5 ± 0.2	2.1 ± 0.0
TPS/30KF	25 ± 2	192 ± 4	720 ± 2	80.6 ± 2.1	39.1 ± 0.5	10.7 ± 0.1	2.1 ± 0.0
TPS/10CF	24 ± 1	168 ± 19	864 ± 34	82.0 ± 7.8	38.8 ± 3.4	6.9 ± 0.2	2.1 ± 0.0
TPS/20CF	23 ± 2	199 ± 8	727 ± 3	87.8 ± 0.1	43.8 ± 0.1	9.9 ± 0.1	2.0 ± 0.0
TPS/30CF	26 ± 1	221 ± 3	644 ± 21	80.8 ± 4.2	43.4 ± 0.8	12.4 ± 0.1	1.9 ± 0.1
TPS/10APP	33 ± 0	235 ± 17	483 ± 14	52.6 ± 1.8	33.6 ± 1.3	23.3 ± 0.3	1.6 ± 0.0
TPS/15APP	33 ± 1	237 ± 17	438 ± 7	48.3 ± 1.1	32.2 ± 0.6	26.3 ± 0.0	1.5 ± 0.0
TPS/20APP	35 ± 1	261 ± 11	395 ± 2	44.0 ± 1.0	31.4 ± 0.3	28.5 ± 0.8	1.4 ± 0.0
TPS/30APP	43 ± 1	267 ± 7	376 ± 4	43.4 ± 0.7	29.6 ± 0.1	34.5 ± 0.2	1.5 ± 0.0
TPS/15KF/15APP	31 ± 1	271 ± 8	379 ± 5	49.9 ± 0.4	33.2 ± 1.5	24.3 ± 3.3	1.5 ± 0.1
TPS/20KF/10APP	31 ± 2	276 ± 12	407 ± 12	54.3 ± 1.6	33.6 ± 1.5	24.1 ± 0.3	1.6 ± 0.0
TPS/10CF/15APP	32 ± 2	240 ± 8	407 ± 1	42.2 ± 0.1	29.3 ± 0.4	30.0 ± 0.2	1.4 ± 0.0
TPS/20CF/10APP	27 ± 2	264 ± 2	338 ± 7	43.1 ± 1.1	29.6 ± 0.3	28.8 ± 0.1	1.5 ± 0.0
